# Quantifying Nonadiabaticity in Major Families of Superconductors

**DOI:** 10.3390/nano13010071

**Published:** 2022-12-23

**Authors:** Evgueni F. Talantsev

**Affiliations:** 1 M. N. Miheev Institute of Metal Physics, Ural Branch, Russian Academy of Sciences, 18 S. Kovalevskoy Str., 620108 Ekaterinburg, Russia; evgeny.talantsev@imp.uran.ru; Tel.: +7-912-676-0374; 2 NANOTECH Centre, Ural Federal University, 19 Mira Str., 620002 Ekaterinburg, Russia

**Keywords:** nonadiabatic effects in superconductors, Heusler alloys, Laves phases, magic-angle twisted bilayer graphene, hydrogen-rich superconductors

## Abstract

The classical Bardeen–Cooper–Schrieffer and Eliashberg theories of the electron–phonon-mediated superconductivity are based on the Migdal theorem, which is an assumption that the energy of charge carriers, $k_{B}T_{F}$, significantly exceeds the phononic energy, $\hbar \omega_{D}$, of the crystalline lattice. This assumption, which is also known as adiabatic approximation, implies that the superconductor exhibits fast charge carriers and slow phonons. This picture is valid for pure metals and metallic alloys because these superconductors exhibit $\frac{\hbar \omega_{D}}{k_{B}T_{F}}<0.01$. However, for *n*-type-doped semiconducting SrTiO_3_, this adiabatic approximation is not valid, because this material exhibits $\frac{\hbar \omega_{D}}{k_{B}T_{F}}≅50$. There is a growing number of newly discovered superconductors which are also beyond the adiabatic approximation. Here, leaving aside pure theoretical aspects of nonadiabatic superconductors, we classified major classes of superconductors (including, elements, A-15 and Heusler alloys, Laves phases, intermetallics, noncentrosymmetric compounds, cuprates, pnictides, highly-compressed hydrides, and two-dimensional superconductors) by the strength of nonadiabaticity (which we defined by the ratio of the Debye temperature to the Fermi temperature, $\frac{T_{\theta }}{T_{F}}$). We found that the majority of analyzed superconductors fall into the $0.025\leq \frac{T_{\theta }}{T_{F}}\leq 0.4$ band. Based on the analysis, we proposed the classification scheme for the strength of nonadiabatic effects in superconductors and discussed how this classification is linked with other known empirical taxonomies in superconductivity.

## 1. Introduction

The majority of experimental works in superconductivity utilize the classical Bardeen–Cooper–Schrieffer (BCS) [1] and Migdal–Eliashberg (ME) [2,3] theories as primary tools to analyze measured data. However, it should be clarified that these theories are valid for superconductors which satisfy the condition designated by the Born–Oppenheimer–Migdal approximation [4]:

(1) $$\frac{\hbar \omega_{D}}{k_{B}T_{F}}=\frac{T_{\theta }}{T_{F}}={\frac{88\;K}{1.1\times 10^{5}\;K}|}_{Pb}=8\times 10^{-4}\ll 1$$

where ℏ is the reduced Planck constant, $\omega_{D}$ is the Debye frequency, $k_{B}$ is the Boltzmann constant, $T_{\theta }$ is the Debye temperature, $T_{F}$ is the Fermi temperature, and data for lead were reported by Poole [5]. The Born–Oppenheimer–Migdal approximation allows the separation of electronic and ionic motions in metals, because Equation (1) implies that the conductor exhibits fast charge carriers (for which characteristic energy scale is related to the Fermi temperature, $T_{F}$) and relatively slow phonons (for which characteristic energy scale is related to the Debye temperature, $T_{\theta }$).

However, Equation (1) satisfies for many, but not for all superconductors, and the first discovered superconductor for which Equation (1) was found to be violated is *n*-type doped semiconducting SrTiO_3_ [6]:

(2) $$\frac{\hbar \omega_{D}}{k_{B}T_{F}}=\frac{T_{\theta }}{T_{F}}={\frac{627\;K}{13\;K}|}_{{\mathrm{SrTiO}}_{3}}=48\gg 1$$

where data for SrTiO_3_ is taken from [7,8]. The theoretical description of the superconductivity in materials, in which the charge carriers and the lattice vibrations exhibit characteristic energy scales similar to Equation (2), is complicated, and the general designation of these superconductors are as nonadiabatic superconductors [9,10,11,12,13,14,15,16]. This theory [9,10,11,12,13,14,15,16] provides a general equation for the superconducting transition temperature, $T_{c}$, in nonadiabatic superconductors [9]: $T_{c}=1.134\times \frac{\varepsilon_{F}}{k_{B}}\times e^{-\frac{1}{\lambda_{nad}}}$, where $\varepsilon_{F}$ is the Fermi energy, and $\lambda_{nad}$ is the coupling strength constant in nonadiabatic superconductors, which serves a similar role to the electron–phonon coupling strength, $\lambda_{e-ph}$, in the BCS [1] and ME [2,3] theories. In addition, one of the primary fundamental theoretical problems is calculating this constant with acceptable accuracy to describe the experiment [4,5,6,7,8,9,10,11,12,13,14,15,16].

For experimentalists, it is important to have a simple practical routine to establish the strength of nonantiadiabatic effects in newly discovered superconductors. The most obvious parameter, which serves as an experimentally measured value to quantify the strength of nonantiadiabaticity, is the $\frac{T_{\theta }}{T_{F}}$ ratio. For practical use of this criterion, there is a need for the taxonomy of possible $\frac{T_{\theta }}{T_{F}}$ values.

To establish the taxonomy, we performed the analysis for a broad a range as possible of superconductors; these range from two- to three-dimensional materials, from elements to compounds of up to five elements, from low-$T_{c}$ (with $T_{c}\sim 0.1\;K$) to record high-$T_{c}$ (with $T_{c}=240\;K$) hydrides, and from materials that exhibit a high order of crystalline lattice symmetry to the materials with low symmetry. Namely, we tried to cover all superconductors for which primary characteristic parameters (apart $T_{c}$, $T_{\theta }$, and $T_{F}$), such as the London penetration depth, λ(0), the coherence length, ξ(0), the amplitude of the superconducting energy gap, Δ(0), and the electron–phonon coupling strength constant, $\lambda_{e-ph}$, were established. In the results, we presented the analysis of more than 40 superconductors within the families of main superconductors.

Based on our analysis, we proposed the following classification scheme:

(3) $$\{\begin{array}{c} \;\;\;\;\;\;\frac{T_{\theta }}{T_{F}}<0.025\to adiabatic\;superconductor;\;\;\;\;\;\;\\ 0.025≲\frac{T_{\theta }}{T_{F}}≲0.4\to moderately\;strong\;nonadiabaticity;\\ \;\;0.4<\frac{T_{\theta }}{T_{F}}\to \;nonadiabatic\;superconductor.\;\;\;\;\;\;\;\;\;\; \end{array}$$

One of our findings is that for weakly nonadiabatic superconductors (i.e., for materials exhibited $0.025\leq \frac{T_{\theta }}{T_{F}}≲0.4$), the predicting power of the BCS-ME theories (for instance, the prediction of the superconducting transition temperature) is reasonably accurate. However, all these superconductors are located outside of the BCS corner in the Uemura plot.

We also showed how the proposed classification scheme is linked to other known empirical scaling laws and taxonomies in superconductivity [13,17,18,19,20,21]; meanwhile, the search for the link of the proposed taxonomy with the recently reported big data [22,23] is under progress.

## 2. Utilized Models

Proposed taxonomy is based on the knowledge of three fundamental temperatures of the superconductor, which are $T_{c}$, $T_{\theta }$, and $T_{F}$. The superconducting transition temperature, $T_{c}$, is directly measured in either temperature resistance or in magnetization experiments. It is also important to mention the primary experimental techniques and theoretical models utilized to deduce the Debye temperature, $T_{\theta }$, and the Fermi temperature, $T_{F}$, in superconductors.

There are two primary techniques to determine the Debye temperature, $T_{\theta }$. One technique is to analyze the measured temperature-dependent normal-state specific heat, $C_{p}\left(T\right)$, from which the electronic specific heat coefficient, $\gamma_{n}$, and the Debye temperature, $T_{\theta }$, are deduced (see, for instance [24,25,26]):

(4) $$\frac{C_{p}\left(T\right)}{T}=\gamma_{n}+\beta T^{2}+\alpha T^{4}$$

where β is the Debye law lattice heat-capacity contribution, and *α* is from higher order lattice contributions. The Debye temperature can be calculated:

(5) $$T_{\theta }={\left(\frac{12\pi^{4}Rp}{5\beta }\right)}^{\frac{1}{3}}$$

where R is the molar gas constant, and *p* is the number of atoms per formula unit.

Another technique is to fit normal-state temperature dependent resistance, *R*(*T*), to the Bloch–Grüneisen (BG) equation [24,25,26,27,28]:

(6) $$R\left(T\right)=\frac{1}{\frac{1}{R_{sat}}+\frac{1}{R_{0}+A\times {\left(\frac{T}{T_{\theta }}\right)}^{5}\times \int_{0}^{\frac{T_{\theta }}{T}}\frac{x^{5}}{\left(e^{x}-1\right)\left(1-e^{-x}\right)}\cdot dx}}$$

where, $R_{sat}$ is the saturated resistance at high temperatures which is temperature independent, $R_{0}$ is the residual resistance at T→0 K, and A is free fitting parameter. Many research groups utilized both techniques (i.e., Equations (4)–(6)) to deduce $T_{\theta }$ [24,25,26,27,29].

From the measured $T_{c}$ and the deduced $T_{\theta }$, one can derive the electron–phonon coupling constant, $\lambda_{e-ph}$, as a root of either the original McMillan equation [30], or its recently revisited form [27]:

(7) $$T_{c}=\left(\frac{1}{1.45}\right)\times T_{\theta }\times e^{-\left(\frac{1.04\left(1+\lambda_{e-ph}\right)}{\lambda_{e-ph}-\mu^{*}\left(1+0.62\lambda_{e-ph}\right)}\right)}\times f_{1}\times f_{2}^{*}$$

(8) $$f_{1}={\left(1+{\left(\frac{\lambda_{e-ph}}{2.46\left(1+3.8\mu^{*}\right)}\right)}^{3/2}\right)}^{1/3}$$

(9) $$f_{2}^{*}=1+\left(0.0241-0.0735\times \mu^{*}\right)\times \lambda_{e-ph}^{2}$$

where $\mu^{*}$ is the Coulomb pseudopotential, $0.10≲\mu^{*}≲0.15$ [27,30].

There are several experimental techniques to derive the Fermi temperature, $T_{F}$, from experimental data. One of these techniques is to measure the temperature dependent Seebeck coefficient, S(T), and fit a measured dataset to the equation [8]:

(10) $$\left|\frac{S\left(T\right)}{T}\right|=\frac{\pi^{2}}{3}\frac{k_{B}}{e}\frac{1}{T_{F}}$$

Another approach is to measure the magnetic quantum oscillations [31], from which the magnitude of charge carrier mass, $m^{*}=m_{e}\left(1+\lambda_{e-ph}\right)$ (where $m_{e}$ is bare mass of electron), together with the size of the Fermi wave vector, $k_{F}$, can be obtained and plugged into [31]:

(11) $$T_{F}=\frac{\hbar^{2}}{2k_{B}}\frac{k_{F}^{2}}{m^{*}}$$

An alternative approach is based on the extraction of the charge carriers mass, $m^{*}$, and density, n, as two of four parameters from the simultaneous analysis of $C_{p}\left(T\right)$, R(T), the muon spin relaxation (*μ*SR), the lower critical field data, $B_{c1}\left(T\right)$, and the upper critical field data, $B_{c2}\left(T\right)$ [32], and plugging these parameters into the equation for an isotropic spherical Fermi surface [32]:

(12) $$T_{F}=\frac{\hbar^{2}}{2k_{B}}\frac{1}{m^{*}}{\left(3\pi^{2}n_{s}\right)}^{\frac{2}{3}}$$

where $n_{s}$ is bulk charge curriers density at T→0 K. For 3D superconductors, $n_{s}$ is given by the equation [33]:

(13) $$n_{s}\left(0\right)=\frac{m^{*}}{\mu_{0}e^{2}}\frac{1}{\lambda^{2}\left(0\right)}$$

where $\mu_{0}$ is the permeability of free space, l is the charge carrier mean free path, λ(0) is the ground state London penetration depth, and ξ(0) is the ground state coherence length.

It should be noted that λ(0) can be also deduced from the ground state lower critical field [28,34]:

(14) $$B_{c1}\left(0\right)=\frac{\phi_{0}}{4\pi }\frac{ln\left(1+\sqrt{2}\kappa \left(0\right)\right)}{\lambda^{2}\left(0\right)}$$

where $\kappa \left(0\right)=\frac{\lambda \left(0\right)}{\xi \left(0\right)}$ is the ground state Ginzburg–Landau parameter.

For two dimensional (2D) superconductors, $T_{F}$ can be determined from *μ*SR measurements and crystallographic data [18]:

(15) $$T_{F}=\frac{\pi \hbar^{2}}{k_{B}}\frac{1}{m^{*}}n_{s}\times c_{int}$$

where $c_{int}$ is the average distance between superconducting planes.

If measuring techniques are limited to the magnetoresistance measurements, R(T,B) (which was the case in the field of highly-compressed near-room temperature superconductors (NRTS) [35,36,37,38,39,40,41,42,43,44,45,46,47,48,49,50], until recent experimental progress by Minkov et al. [51,52]), $T_{F}$ can be estimated by the equation [53]:

(16) $$T_{F}=\frac{\pi^{2}m^{*}}{2k_{B}\hbar^{2}}\times \xi^{2}\left(0\right)\times \Delta^{2}\left(0\right)$$

where Δ(0) is the ground state amplitude of the superconducting energy gap, which is varying in a reasonably narrow range $3.2\leq \frac{2\Delta \left(0\right)}{k_{B}T_{c}}\leq 5.0$, so that the ballpark value for $T_{F}$ can be estimated. For instance, ξ(0) can be deduced from magnetoresistance measurements [54] and the electron–phonon coupling strength constant, $\lambda_{e-ph}$, can be assumed to be the average value of values calculated by first-principles calculations [55,56].

## 3. Results

In Table 1, we present data for major groups of superconductors, where data sources for $T_{c}$, $T_{\theta }$, $T_{F}$, and other parameters (for instance, $\lambda_{e-ph}$) are given. 

**Figure 1:**
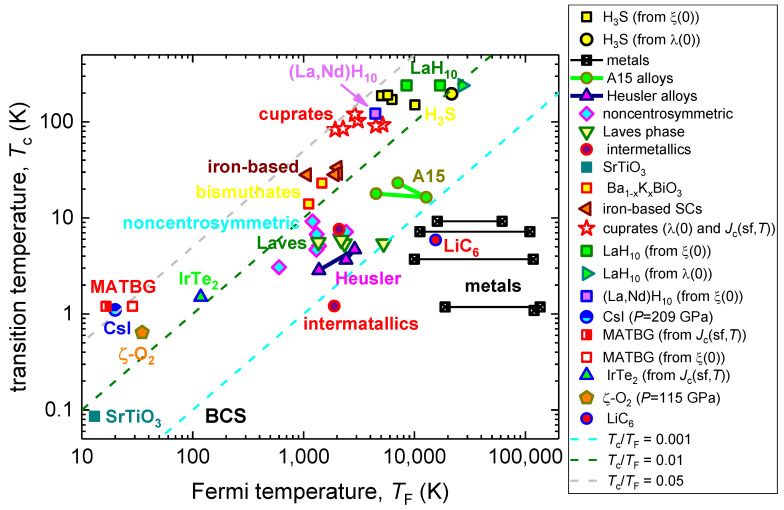
Uemura plot (*T_c_* vs. *T_F_*) for primary superconducting families. References on original data (*T_c_* and *T_F_*) can be found in Table 1.

In Figure 1, we show the $T_{c}\;\mathrm{vs}.T_{F}$ dataset in a log–log plot, which is the traditional data representation in the well-known Uemura plot [18].

**Figure 2:**
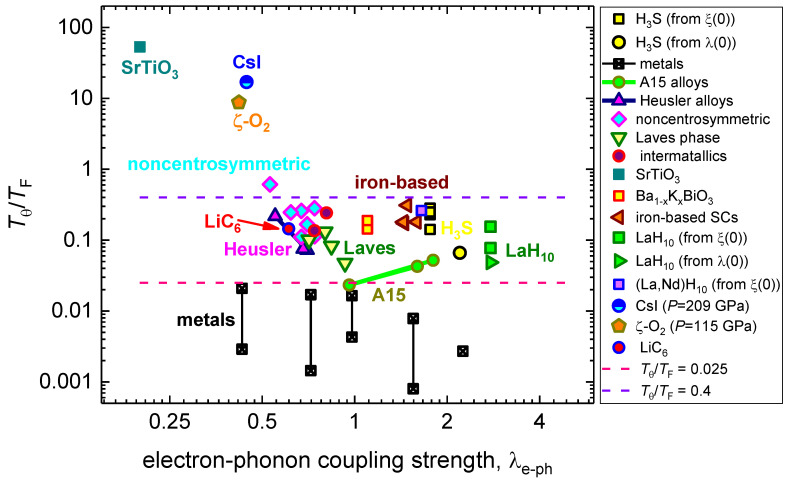
Plot of $\frac{T_{\theta }}{T_{F}}\;\mathrm{vs}.\;\lambda_{e-ph}$ for primary superconducting families. This type of plot proposed by Pietronero et al. [13]. References for original data (*T_θ_*, $\lambda_{e-ph}$, *T_F_*) can be found in Table 1.

In Figure 2, we represent the same superconducting materials, but here we display the $\lambda_{e-ph}\;\mathrm{vs}.\frac{T_{\theta }}{T_{F}}$ dataset in a semi-log plot. To our best knowledge, the $\lambda_{e-ph}\;\mathrm{vs}.\frac{T_{\theta }}{T_{F}}$ plot was first plotted by Pietronero et al. [13] in linear–linear scales. However, because the $\frac{T_{\theta }}{T_{F}}$ ratio for main families of superconductors is varied within four orders of magnitude (Table 1), and $0.4\leq \lambda_{e-ph}\leq 3.0$, it is more suitable to use the semi-log plot (Figure 2).

**Figure 3:**
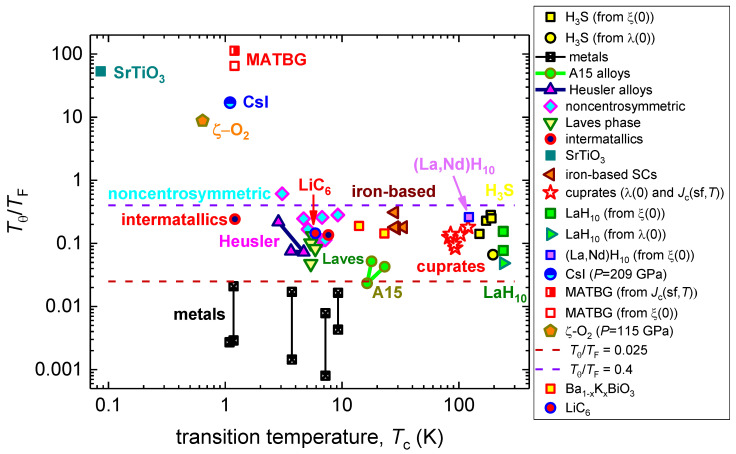
Plot $\frac{T_{\theta }}{T_{F}}\;\mathrm{vs}.\;T_{c}$ for primary superconducting families. References on original data (*T*_θ_, *T_c_*, *T_F_*) can be found in Table 1.

Finally, in Figure 3, we represented the same superconducting materials, but here we displayed the $T_{c}\;\mathrm{vs}.\frac{T_{\theta }}{T_{F}}$ dataset in a log–log plot. This type of plot was chosen because as $T_{c}$, as $\frac{T_{\theta }}{T_{F}}$ are varied within several orders of magnitude.

## 4. Discussion

The family of near-room temperature superconductors (NRTS) is represented in Table 1 and Figure 1 by H_3_S (*P* = 155 GPa), SnH_12_ (*P* = 190 Gpa), and La_1-x_Nd_x_H_10_ (x = 0.09, *P* = 180 Gpa). Two independent approaches were used to perform calculations in H_3_S:

1. $T_{F}$ was calculated based on Equations (12) and (13). In these calculations λ(0)=37 nm (extracted from the analysis of DC magnetization experiments reported by Minkov et al. [51,52]) was used.
2. $T_{F}$ was calculated based Equations (6)–(9) and (18), in which utilized ξ(0) values were extracted [53] from magnetoresistance measurements reported by Mozaffari et al. [54]).

It should be noted that, in both approaches, the electron–phonon coupling strength constant, $\lambda_{e-ph}$, was assumed to be $\lambda_{e-ph}=1.76$, which is the average value of values calculated by first-principles calculations [55,56], and values extracted from experimental *R*(*T*) data [27].

It can be seen in Table 1 and Figure 1 that the calculated $T_{F}$ values for H_3_S, by two alternative approaches, are in a very good agreement with each other. To demonstrate the acceptable level of variation in $T_{F}$ values for the same material, in Table 1 and Figure 1 we present the results of the calculations for pure metals, where $T_{F}$ was calculated by the two approaches mentioned above and the use of experimental data reported by different research groups.

$T_{F}$ in HTS cuprates were calculated by the Equations (13) and (15), which do not require the knowledge of the electron–phonon coupling constants, $\lambda_{e-ph}$. This is despite Ledbetter et al. [7] reporting the so-called effective electron–phonon coupling strength, $\lambda_{e-ph,eff}$, from which the effective mass can be deduced, $m^{*}=\left(1+\lambda_{e-ph,eff}\right)\times m_{e}$.

In addition, it should be noted that for YBa_2_Cu_3_O_7_, Uemura [83] reported the relation [83]:

(17) $$\frac{m^{*}}{m_{e}}=2.5$$

from which $\lambda_{e-ph}=1.5$ can be derived. Calculated values are in a reasonable agreement with experimental $\frac{m^{*}}{m_{e}}$ values reported by several research groups [108,109,110] in YBa_2_Cu_3_O_7-x_.

However, because the phenomenology of the electron–phonon mediated superconductivity cannot describe the superconducting state in cuprates, and the $T_{\theta }$ for cuprates were taken as experimental values (see, for instance, report by Ledbetter et al. [7,88]), all cuprate superconductors are shown in Figure 1 and Figure 3 and are not shown in Figure 2.

It should be mentioned that the result of the $T_{F}$ calculation in MATBG (Table 1), $T_{F}=16.5\;K$, which was primarily based on the London penetration depth, λ(0)=1860 nm, was deduced in Ref. [96] from the self-field critical current density, $J_{c}\left(sf,T\right)$, by the approach proposed by us [84]:

(18) $$J_{c}\left(sf,T\right)=\frac{\phi_{0}}{4\pi \mu_{0}}\frac{ln\left(1+\sqrt{2}\kappa \left(T\right)\right)}{\lambda^{3}\left(T\right)}$$

The remarkable agreement of the deduced value, $T_{F}=16.5\;K$, and the value reported in the original work on MATBG by Cao et al. [95], $T_{F}=17\;K$, which was calculated based on normal state charge carriers density in MATBG, independently validates our primary idea [84] about the fundamental nature of the self-field critical current in weak-links samples [84,85,111]. This concept was recently proven by Paturi and Huhtinen [112], who utilized the fact that the London penetration depth, λ(0), in real samples, depends on the mean free-path of charge carriers, l:

(19) $$\lambda \left(0\right)=\lambda_{clean\;limit}\left(0\right)\sqrt{1+\frac{\xi \left(0\right)}{l}}$$

where λ(0) is the effective penetration depth, and $\lambda_{clean\;limit}\left(0\right)$ is the penetration depth in samples, exhibiting a very long mean free-path, l≫ξ(0). Paturi and Huhtinen [112] varied l in YBa_2_Cu_3_O_7-x_ films and showed that the change in $J_{c}\left(st,T\right)$ satisfies Equations (18) and (19).

Materials, in which λ(0) was deduced by the mean temperature dependent self-field critical current density, $J_{c}\left(sf,T\right)$ (Equation (18)), have designation “$J_{c}\left(sf,T\right)$” in Figure 1, Figure 2 and Figure 3.

The MATBG does not show in Figure 2, because the derivation of $\lambda_{e-ph}$ cannot be performed by the used phenomenology: $m^{*}=\left(1+\lambda_{e-ph}\right)\times m_{e}$, because $\frac{m^{*}}{m_{e}}=0.2$ [96]; however, this material is shown in Figure 1 and Figure 3, because $\lambda_{e-ph}$ is not required for these plots.

Returning back to hydrides, we need to note that Durajski [56] performed first-principles and studied the strength of the nonadiabatic effects in highly-compressed sulfur hydride and phosphorus hydride. Calculations show that the strength of the nonadiabatic effects can be quantified as moderately weak in comparison with the classical nonadiabatic superconductor SrTiO_3_. This is in a good agreement with our result (see Figure 3 and Table 1), that all deduced $\frac{T_{\theta }}{T_{F}}$ values for NRTS are within the range of:

(20) $$0.03\leq \frac{T_{\theta }}{T_{F}}\leq 0.3$$

Moreover, the classical nonadiabatic superconductor SrTiO_3_ falls into the intermediate zone between unconventional and BCS superconductors; this is because this material exhibits $\frac{T_{c}}{T_{F}}=0.0066$, and by this criterion, SrTiO_3_ is similar to the Laves phase materials, intermetallics, A-15 alloys, and Heusler alloys, which cannot be considered to be a correct manifestation of primary uniqueness for this nonadiabatic material.

More unexpectedly, a two dimensional LiC_6_ (which is a lithium-doped graphene) superconductor falls into the BCS metals zone in the Uemura plot (Figure 1), despite the fact that this material exhibits reasonable strength in the nonadiabatic effects, $\frac{T_{c}}{T_{F}}=0.15$ [99].

However, in Figure 2 and Figure 3, the outstanding separations of all nonadiabatic superconductors from their adiabatic and moderate nonadiabatic counterparts are clearly manifested.

By looking at the data in Figure 2 and Figure 3, it is easy to recognize that ¾ (32 of 42) of the analyzed superconductors fall into a reasonably narrow band:

(21) $$0.025\leq \frac{T_{\theta }}{T_{F}}\leq 0.4$$

Based on this, we proposed that the values in Equation (21) were used as empirical limits for the adiabatic superconductors ($\frac{T_{\theta }}{T_{F}}\leq 0.025$), moderate nonadiabatic superconductors ($0.025\leq \frac{T_{\theta }}{T_{F}}\leq 0.4$), and strong nonadiabatic superconductors ($\frac{T_{\theta }}{T_{F}}\geq 0.4$).

It also follows from our analysis that all strong nonadiabatic superconductors exhibit low superconducting transition temperatures, $T_{c}\leq 1.2\;K$ (Figure 3).

## 5. Conclusions

In this work, we proposed a new classification scheme to quantify the effects of nonadiabaticity in superconductors. By performing the analysis of experimental data for more than 40 superconductors, which represent the primary families of superconductors, we found that ¾ of all analyzed superconductors fall into a narrow $0.025\leq \frac{T_{\theta }}{T_{F}}\leq 0.4$ band. Based on this, we proposed the taxonomy for the strength of the nonadiabatic effects in superconductors.

## Figures and Tables

**Table 1:** Superconductors and their parameters used in the work. In all calculations (except some original sources, $\mu^{*}=0.13)$.

Type/Chemical Composition	λ(0) (nm)	ξ(0) (nm)	λ_e-ph_	*T*_c_ (K)	*T*_θ_ (K)	$\frac{2\Delta \left(0\right)}{k_{B}T_{c}}$	*T*_F_ (10^3^ K)	*T*_θ_ */T*_F_
**Pure metals**								
Aluminium				1.18 [57,58]	394 [5]		136 [5]	$2.9\times 10^{-3}$
Aluminium	50 [57]	1550 [58]	0.43 [59]	1.18 [57,58]	394 [5]	3.535 [59]	18.9 (Equation (12))	$2.1\times 10^{-2}$
Tin				3.72 [58]	170 [5]		118 [5]	$1.4\times 10^{-3}$
Tin	77 [60]	180 [58]	0.72 [59]	3.72 [58]	170 [5]	3.705 [59]	10.0 (Equation (12))	$1.2\times 10^{-2}$
Lead				7.20 [58]	88 [5]		110 [5]	$8\times 10^{-4}$
Lead	64 [60]	87 [58]	1.55 [59]	7.20 [60]	88 [5]	4.497 [59]	11.2 (Equation (12))	$7.8\times 10^{-3}$
Niobium				9.25 [58]	265 [5]		61.8 [5]	$4.3\times 10^{-3}$
Niobium	52 [58]	39 [58]	0.98 [59]	9.25 [58]	265 [5]	3.964 [59]	16.1 (Equation (12))	$1.6\times 10^{-2}$
Gallium	52 [58]	39 [58]	2.25 [59]	1.09 [5,32,58,59]	325 [5,32,58,59]		120 [5,32,58]	$2.7\times 10^{-3}$
**A15 Alloys**								
Nb_3_Sn	124 [61]	3.6 [61]	1.8 [62]	17.9 [61]	234 [61]	4.2 [62]	4.5 (Equation (12))	$5.2\times 10^{-2}$
V_3_Si	62 [62]	3.3 [62]	0.96 [62]	16.4 [63]	297 [64]	3.7 [62]	12.8 (Equation (12))	$2.3\times 10^{-2}$
Nb_3_Ge	90 [58]	3.0 [58]	1.60 [59]	23.2 [58]	302 [65]	4.364 [59]	7.1 (Equation (12))	$4.3\times 10^{-2}$
**Heusler alloys**								
ZrNi_2_Ga	350 [66]	15 [66]	0.551 [66]	2.85 [66]	300 [66]		1.4 (Equation (12))	$2.2\times 10^{-1}$
YPd_2_Sn	196 [67]	19 [67]	0.70 [67]	4.7 [67]	210 [67]	4.1 [67]	2.9 (Equation (12))	$7.2\times 10^{-2}$
HfPd_2_Al	225 [67]	13 [67]	0.68 [67]	3.66 [67]	182 [67]	3.74 [67]	2.4 (Equation (12))	$7.5\times 10^{-2}$
**Noncentrosymmetric**								
Nb_0.5_Os_0.5_	654 [68]	7.8 [68]	0.53 [68]	3.07 [68]	367 [68]	3.62 [68]	0.60 (Equation (12))	$6.1\times 10^{-1}$
Re_6_Zr (mSR)	356 [29]	3.7 [29]	0.67 [29]	6.75 [29]	338 [29]	3.72 [29]	1.3	$2.6\times 10^{-1}$
Re_6_Zr (magnetization)	247 [29]	3.3 [29]	0.67 [29]	6.75 [29]	237 [29]	3.72 [29]	2.1	$1.1\times 10^{-1}$
Mo_3_Al_2_C	376 [69]	4.2 [69]	0.74 (Equations (7)–(9))	9.2 [69]	339 [69]	4.03 [69]	1.2	$2.8\times 10^{-1}$
NbIr_2_B_2_ [70]	223	4.5	0.74	7.18	274		2.4	$1.1\times 10^{-1}$
TaIr_2_B_2_ [70]	342	4.7	0.70	5.1	230		1.4	$1.7\times 10^{-1}$
Re_3_Ta [71]			0.62	4.7	321		0.64	$5.0\times 10^{-1}$
**Laves phases**								
BaRh_2_ [72]	340	8.4	0.80	5.6	178		1.4	$1.3\times 10^{-1}$
SrRh_2_ [72]	229	9.1	0.71	5.4	237		2.3	$1.0\times 10^{-1}$
SrRh_2_ [73]	121	8.6	0.93	5.4	250		5.3	$4.7\times 10^{-2}$
SrIr_2_ [74]	237	7.5	0.84	5.9	180		2.3	$8.2\times 10^{-2}$
**Intermetallics**								
MgCNi_3_ [75]	248	4.6	0.74 (Equations (7)–(9))	7.6	284		2.1	$1.4\times 10^{-1}$
RuAl_6_ [76]	265	27.7	0.81	1.21	458		1.9	$2.4\times 10^{-1}$
**Perovskite**								
SrTiO_3_			0.2 [7]	0.086 [8]	690 [77]		$1.3\times 10^{-2}$ [8]	$5.3\times 10^{1}$
**Pnictides**								
ThFeAsN	375 [78]		1.48 [78]	28.1 [78]	332 [79]		0.47 (Equation (17)) $c_{int}=8.5\;Å$ [78]	$7.0\times 10^{-1}$
KCa_2_Fe_4_As_4_F_2_	230 [80]		1.59 [80]	33.4 [80]	366 [80]		1.3 (Equation (17)) $c_{int}=8.5\;Å$ [80]	$2.9\times 10^{-1}$
RbCa_2_Fe_4_As_4_F_2_	232 [80]		1.45 [80]	29.2 [80]	332 [80]		1.2 (Equation (17)) $c_{int}=8.5\;Å$ [80]	$2.8\times 10^{-1}$
CsCa_2_Fe_4_As_4_F_2_	244 [80]		1.44 [80]	28.3 [80]	344 [80]		1.1 (Equation (17)) $c_{int}=8.5\;Å$ [80]	$3.1\times 10^{-1}$
**Cuprates**								
YBa_2_Cu_3_O_7_ [81]	115 [81,82]	2.5 [81]	1.5 [83]	93.2 [81]	437 [7]		3.4 (Equation (17)) $c_{int}=5.8\;Å$ [83]	$1.2\times 10^{-1}$
(Y,Dy)Ba_2_Cu_3_O_7_ [84]	128 [84,85]	2.5 [81]	1.5 [83]	90.4 [84,85]	437 [7]	4.24 [84,85]	2.9 (Equation (17)) $c_{int}=5.8\;Å$ [83]	$1.5\times 10^{-1}$
Bi_2_Sr_2_CaCu_2_O_8_ [86]	196 [85]	1.2 [85]	4.7 [7]	82.7 [85]	240 [7]	3.9 [85]	1.2 (Equation (17)) $c_{int}=6\;Å$ [83]	$2.0\times 10^{-1}$
Tl_2_Ba_2_CaCu_2_O_8_ [87]	179 [85]	1.2 [85]		103 [85]	425 [88]	4.3 [85]	1.5 (Equation (17)) $c_{int}=6\;Å$ [83]	$2.9\times 10^{-1}$
HgBa_2_CaCu_2_O_8_ [89]	188 [85]	1.6 [85]		120 [85]	525 [88]	3.3 [85]	1.3 (Equation (17)) $c_{int}=6\;Å$ [83]	$3.9\times 10^{-1}$
Bi_2_Sr_2_Ca_2_Cu_3_O_10_ [90]	175 [85]	1.0 [85]	4.5	85 [85]	319 [7]	4.5 [85]	1.5 (Equation (17)) $c_{int}=6\;Å$ [83]	$2.1\times 10^{-1}$
**Bismuthates**								
Ba_1-x_K_x_BiO_3_ (x = 0.4) [91,92]			1.10 [93]	23 [92]	210 [94]	3.8-4.1 [93]	1.5 [92]	$1.4\times 10^{-1}$
Ba_1-x_K_x_BiO_3_ (x = 0.5) [91,92]			1.10 [93]	14 [92]	210 [94]	3.8-4.0 [93]	1.1 [92]	$1.9\times 10^{-1}$
**2D superconductors**								
MATBG [95]	2180 [96]		$\frac{m^{*}}{m_{e}}=0.2$ [96]	1.2 [96]	1864 [97]	4.4 [96]	$16.5\times 10^{-3}$ (Equation (15)) $c_{int}=1\;\mathrm{nm}$	$1.1\times 10^{2}$
MATBG [95]		61.4 [96]	[96]	1.2 [96]	1864 [97]	4.4 [96]	$28.6\times 10^{-3}$ (Equation (16))	$6.5\times 10^{1}$
Li-doped graphene, LiC_6_ [98]			0.61 [99]	5.9 [98]	2240 [99]		15.5 [99]	$1.45\times 10^{-1}$
IrTe_2_ [100] (sample thickness is 21 nm)	600 [100]	75 [100]		1.6 [100]		5.46 [100]	0.118 (Equation (15)) $c_{int}=0.54\;\mathrm{nm}$	
**Ionic Salt**								
CsI (*P* = 206 GPa) [101]			0.445 [102]	1.1 [101]	339 [102]		$\left(20\pm 4\right)\times 10^{-2}$ [102]	17±4 [102]
**NRTS hydrides**								
H_3_S (*P* = 155 GPa) [35]	37 [52]	1.9 [54]	2.2 [103]	197 [103]	1427 [103]		21.6 (Equation (12)) and [104]	$6.6\times 10^{-2}$
H_3_S (*P* = 155 GPa) [35]		1.9 [54]	1.76 [55,56]	197 [103]	1427 [103]	3.55 [53]	10±3 (Equation (16)) and [104]	$\left(1.4\pm 0.3\right)\times 10^{-1}$
LaH_10_ (*P* = 150 GPa) [36]	30 [51]	1.5 [51]	2.77 [27]	240 [27]	1310 [27]		27.0 (Equation (12))	$2.7\times 10^{-2}$
La_1-x_Nd_x_H_10_ (x = 0.15) (*P* = 180 GPa) [48]		2.3 [105]	1.65 [105]	122 [105]	1156 [105]	4.0 [105]	4.4 [105] (Equation (16))	$2.6\times 10^{-1}$
**Compressed oxygen**								
ζ-O_2_ (*P* = 115 GPa) [106]		42 [107]	0.42 [107]	0.64 [107]	306 [107]		$3.5\times 10^{-2}$ [107] (Equation (16))	8.7

## Data Availability

Not applicable.
